# Altered Nasal Microbiota in Sinonasal Tumors: A Comparative Analysis of Malignant and Benign Sinonasal Tumors

**DOI:** 10.1002/alr.70123

**Published:** 2026-02-20

**Authors:** Evan A. Patel, Phillip A. Engen, Glen D. Souza, Sarah Khalife, Donyea Moore, Lauren Kret, Pedro Escobedo, Stefan J. Green, Ankur Naqib, Peter Filip, Peter Papagiannopoulos, Bobby A. Tajudeen, Mahboobeh Mahdavinia, Pete S. Batra

**Affiliations:** ^1^ Rush Medical College Rush University Medical Center Chicago Illinois USA; ^2^ Rush Center for Integrated Microbiome and Chronobiology Research Rush University Medical Center Chicago Illinois USA; ^3^ Department of Otorhinolaryngology‐Head and Neck Surgery Rush University Medical Center Chicago Illinois USA; ^4^ Division of Otolaryngology, Head & Neck Surgery Department of Surgery, McMaster University Hamilton Ontario Canada; ^5^ Allergy & Immunology Division, Department of Internal Medicine and Department of Pediatrics University of Texas Health Houston Texas USA; ^6^ Department of Internal Medicine Indiana University School of Medicine Indianapolis Indiana USA; ^7^ Genomics and Microbiome Core Facility Rush University Medical Center Chicago Illinois USA; ^8^ Department of Internal Medicine Rush University Medical Center Chicago Illinois USA; ^9^ Rush Research Bioinformatics Core Rush University Medical Center Chicago Illinois USA

**Keywords:** bacteria, microbes, microbiome, sinonasal malignancy, sinonasal tumors

## Abstract

**Background:**

Although shifts in nasal microbiota have been well‐documented in inflammatory upper airway conditions, microbiota tumor‐associated alterations remain uncharacterized. This study is the first to compare sinonasal microbiota profiles of patients with malignant tumors (MT), benign tumors (BT), and controls, offering insights into tumor‐associated microbiomes.

**Methods:**

This prospective, cross‐sectional, observational study assessed intraoperative sinus swabs from 70 adult research participants (MT = 23, BT = 15, control = 32). Sinonasal microbial communities were characterized using 16S rRNA gene amplicon sequencing to determine if microbial community structures differed between groups.

**Results:**

Tumor‐associated sinonasal microbiota profiles showed clear dysbiosis, with reduced relative abundance of beneficial microbes and increased putative pathogenic taxa. Both MT and BT had significantly lower microbial diversity and distinct compositions compared to controls. MT samples had significantly higher relative abundance of Firmicutes and reduced relative abundance of Actinobacteria. These phylum‐level alterations were accompanied by elevated proinflammatory microbial taxa, paired with reduced relative abundance of keystone, beneficial taxa consistent with healthy nasal microbiomes. Microbial communities in BT and MT samples were similar, but *Alcaligenes* was more abundant, and *Corynebacterium* was less abundant in MT than in BT.

**Conclusion:**

This study observed that sinonasal microbial communities in MT exhibited marked dysbiosis with a reduction in the relative abundance of putative sinonasal commensal taxa compared to controls. These alterations were present to a lesser extent in BT. Future investigations should aim to determine whether these microbial shifts contribute to tumor development or represent secondary effects, with an aim to quantify their impact on outcomes and guide therapeutic strategies.

## 1 Introduction

The human sinonasal cavity harbors a diverse and dynamic microbial community that plays a critical role in mucosal immunity and epithelial homeostasis [1]. Disruptions in these microbial communities, often referred to as dysbiosis, have been implicated in the pathogenesis of a variety of upper airway inflammatory diseases, including chronic rhinosinusitis (CRS), allergic rhinitis, and nasal polyposis [2]. These findings have shifted the traditional paradigm of sinonasal disease from one centered on infection alone to one that incorporates the ecological balance of the host and microbial factors. However, despite growing interest in the role of microbiome in airway health and disease, little is known about the composition and function of the sinonasal microbiota in the setting of neoplastic diseases of the nose and paranasal sinuses [3].

Malignancies of the sinonasal cavity are rare but aggressive, often with a complex and protracted clinical course [4]. While environmental exposures such as wood dust and industrial chemicals have been associated with an increased risk of sinonasal malignancies, the underlying mechanisms of tumor initiation and progression remain poorly understood [5]. In recent years, interest has grown in the potential role of the microbiome as a contributor to oncogenesis, with compelling evidence emerging from gastrointestinal, genitourinary, and oropharyngeal cancers [6, 7, 8]. In colorectal cancer, for instance, the species *Fusobacterium nucleatum* has been implicated in promoting tumor growth and immune evasion, while the species *Helicobacter pylori* remains a well‐established carcinogen in gastric cancer [9, 10]. These associations raise the question of whether analogous microbiota–tumor interactions might exist in the sinonasal tract.

To date, studies investigating sinonasal microbiota in the context of tumors are exceedingly rare. The majority of microbiome research in the upper airway has focused on chronic inflammatory conditions, with findings suggesting that reduced microbial diversity and specific taxa alterations, such as *Staphylococcus aureus* and *Pseudomonas aeruginosa*, may contribute to disease persistence or severity [11]. Whether similar microbial shifts occur in the setting of sinonasal tumors, and whether these changes differ between benign and malignant entities, remains an important area of inquiry. The possibility that microbial dysbiosis could either reflect a tumor‐permissive environment or actively contribute to tumor development warrants further investigation.

Moreover, the interplay between sinonasal tumors and the local microbiota may hold implications for host immunity, tumor progression, and even therapeutic outcomes. Tumors can alter the microenvironment through local inflammation, tissue remodeling, hypoxia, and immune suppression; all of which may reshape microbial communities [12]. Conversely, certain microbial taxa may influence tumor behavior through mechanisms such as immune modulation, genotoxic metabolite production, and biofilm formation [13]. A better understanding of these interactions could yield novel biomarkers for diagnosis, help predict prognosis, and identify new therapeutic targets.

In this study, we present the first comparative analysis of the sinonasal microbiota in patients with malignant tumors, benign tumors, and controls using high‐throughput 16S rRNA gene amplicon sequencing. We hypothesized that sinonasal tumors would be associated with reduced microbial diversity and distinct compositional profiles compared to non‐tumor controls. We further aimed to identify specific taxa enriched in the tumor‐associated microbiota, including potential pathogens, and to determine whether the degree of dysbiosis could differentiate benign from malignant tumors. By characterizing these microbiota profiles, we seek to lay the groundwork for future studies exploring causal relationships and therapeutic implications of microbial shifts in sinonasal tumor biology.

## 2 Methods

### 2.1 Study Design and Patient Population

This prospective, cross‐sectional, observational study was conducted at the Rush University Medical Center after approval by the institutional review board. Informed consent was obtained from all participants prior to sample collection. Adult patients (≥18 years old) scheduled to undergo endoscopic sinus or skull base surgery were recruited and stratified into three cohorts: (i) patients with histologically confirmed malignant sinonasal tumors (MT), (ii) patients with benign sinonasal tumors (BT), and (iii) controls undergoing surgery for endoscopic access to the pituitary gland. All patients had no clinical or endoscopic evidence of chronic rhinosinusitis or sinonasal tumor. Exclusion criteria included recent (within 30 days) use of systemic antibiotics, current or recent sinonasal infection, known immunodeficiency, or a history of sinonasal surgery within the past 6 months. Viral testing for human papillomavirus (HPV) or Epstein–Barr virus (EBV) was not performed for this cohort, as these analyses were outside the scope of the current microbiome‐focused study.

### 2.2 Sample Collection and Preservation

Intraoperative tissue specimens were collected under sterile conditions by the attending surgeon at the start of the case, prior to pre/peri‐operative antibiotic administration. Samples were collected using sterile flocked swabs directly from tumor masses in MT and BT patients or from the middle meatus or sphenoethmoid recess in controls. Swabs were immediately placed into a nucleic acid stabilization buffer to preserve nucleic acids. All samples were stored at −80°C until nucleic acid extraction.

### 2.3 Microbiome Characterization

Genomic DNA was extracted from flash‐frozen nasal swabs using bead‐beating and automated purification using a chemagic DNA Stool 200 Kit H96 (Revvity, Hamburg, Germany) implemented on a Chemagic 360 device. Swabs were placed in ZR BashingBead Lysis Tubes (S6012‐50; Zymo Research) with 1 mL lysis buffer and 20 µL proteinase K from the Chemagic DNA stool kit. Samples were subject to thermal mixing at 70°C for 10 min, followed by bead‐beating using a TissueLyzer II device (Qiagen) set at 30 Hz for 5 min. A 2‐min rest was provided between two sequential bead‐beating episodes. A 5‐min incubation at 95°C was performed before final purification on the Chemagic 360 instrument, according to the manufacturer's instructions.

Genomic DNA was PCR amplified with primers sIDTP5_27Fmix and sIDTP7_338R targeting the V1 to V2 region of microbial small subunit ribosomal RNA genes (Table 1) using a two‐stage PCR amplification protocol described previously [14, 15]. First‐stage PCR amplifications were performed in 10 µL reactions in 96‐well plates, using repliQa HiFi ToughMix (Quantabio). PCR conditions were 98°C for 2 min, followed by 28 cycles of 98°C for 10 s, 57°C for 1 s, and 68°C for 1 s. Subsequently, a second PCR amplification was performed to incorporate barcodes and sequencing adapters, as described previously (e.g., Aboushaala et al., 2024). Pooled PCR products were sequenced on an Illumina NovaSeq6000 instrument using a 500‐cycle SP flow cell with read lengths extended to 2×259 bases. Library preparation, pooling, and MiniSeq sequencing were performed at the Genomics and Microbiome Core Facility (GMCF) at Rush University. NovaSeq sequencing was performed at the DNA Services Facility at the Roy J. Carver Biotechnology Center at the University of Illinois at Urbana‐Champaign.

**TABLE 1:** sIDTP5_27Fmix and sIDTP7_338R primer sequences.

**sIDTP5_27F‐YM**
CTACACGACGCTCTTCCGATCTAGAGTTTGATYMTGGCTCAG
**sIDTP5_27F‐Chl**
CTACACGACGCTCTTCCGATCTAGAATTTGATCTTGGTTCAG
**sIDTP5_27F‐Bor**
CTACACGACGCTCTTCCGATCTAGAGTTTGATCCTGGCTTAG
**sIDTP5_27F‐Bif**
CTACACGACGCTCTTCCGATCTAGGGTTCGATTCTGGCTCAG
**sIDTP5_27F‐Ato**
CTACACGACGCTCTTCCGATCTAGAGTTCGATCCTGGCTCAG
The 27F‐mix is a primer set composed of the primers in a 4:1:1:1:1 ratio. The underlined region represents a linker compatible with IDT unique dual index adapters.
**sIDTP7_338R**
CAGACGTGTGCTCTTCCGATCTGCTGCCTCCCGTAGGAGT

### 2.4 Bioinformatics and Taxonomic Classification

Raw sequencing data were processed with QIIME2 (v2023.5) [16], as described previously (e.g., Aboushaala et al., 2024). Briefly, paired‐end reads were merged with PEAR [17], filtered and denoised via DADA2 [18], primer removal was performed with Cutadapt [19], and taxonomy was assigned using the q2‐feature classifer with a naïve Bayes classifier trained on the SILVA 138 99% OTUs full‐length sequences [20, 21]. Bacterial contaminants were identified and removed using the *decontam* software package based on the prevalence in negative controls [22]. Additionally, host‐associated taxa, like chloroplasts and mitochondria, were removed from the dataset.

Microbial community structure and diversity were analyzed using *q2‐diversity*. To compare microbial community structures between groups, permutational multivariate analysis of variance (PERMANOVA; 9999 permutations) and permutational analysis of multivariate dispersions (PERMDISP; 9999 permutations) were conducted, both using Aitchinson distance [23, 24]. Centroid‐based nonmetric multi‐dimensional scaling (NMDS) plots (vegan package, *R*) were used to visualize the microbial community differences. Both alpha‐diversity and NMDS plots were calculated from amplicon sequence variance (ASV) counts rarefied at 40,000 sequences.

Differentially abundant taxa were identified using center log‐ratio Kruskal–Wallis (CLR‐KW) for group‐wise comparisons. Microbial features with relative abundances below 0.1% were excluded from differential abundance analyses. The *p*‐values were adjusted for multiple comparisons using the Benjamini–Hochberg (BH) method. Microbial features of importance were assessed using the machine‐learning *Boruta* random forest algorithm. Log2 fold‐change (Log2FC) values were calculated for taxa exhibiting significant differential abundance.

Descriptive statistics were conducted and visualized with GraphPad Prism 10.0 (GraphPad Software, San Diego, California, USA). Alpha‐diversity indices were tested for normality (Shapiro–Wilk), followed by one‐way analysis of variance (ANOVA) or Kruskal–Wallis tests based on distribution.

## 3 Results

A total of 70 adult patients were included in the final analysis: 32 controls, 15 BT patients, and 23 MT patients. Clinical data, including demographic characteristics, surgical indication, tumor pathology, and perioperative antibiotic exposure, were collected via chart review and recorded in a deidentified database. The mean age was comparable across groups (controls: 57.9 ± 9.1 years; BT: 55.2 ± 13.7 years; MT: 61.3 ± 10.4 years), and there were no statistically significant differences in sex distribution or history of recent antibiotic use. All participants tolerated sampling without complications, and high‐quality sequencing data were obtained for 100% of swab specimens.

All BT samples included in this study were histologically confirmed inverted papillomas. The study cohort demonstrated histologic diversity, with squamous cell carcinoma representing nearly half of the cases (47.8%). Additional malignancies included olfactory neuroblastoma (17.4%), neuroendocrine carcinomas (8.7%), sarcomas (8.7%), and a range of less frequent tumor types such as adenocarcinoma, mucosal melanoma, sinonasal undifferentiated carcinoma, and chordoma (each 4.3%). This distribution highlights the heterogeneity of sinonasal malignancies represented in the cohort, and reduces the likelihood that observed differences in sinonasal microbial community structure are attributable to overrepresentation of a single tumor histology. Data regarding tobacco use and prior radiation exposure were not collected in this study. Participants with known or endoscopic evidence of chronic rhinosinusitis or acute sinonasal infection were excluded at the time of enrollment to control for underlying inflammatory disease that could influence microbial composition. Given the possibility of post‐obstructive secretions caused by the presence of the tumor, samples were collected from on or as close to the tumor surface as possible to more accurately reflect the tumor milieu and not from a distant location. HPV and EBV status were not assessed in any of the participants.

### 3.1 Comparative Analysis of Sinonasal Microbiota in Malignant and Benign Tumors

**FIGURE 1:**
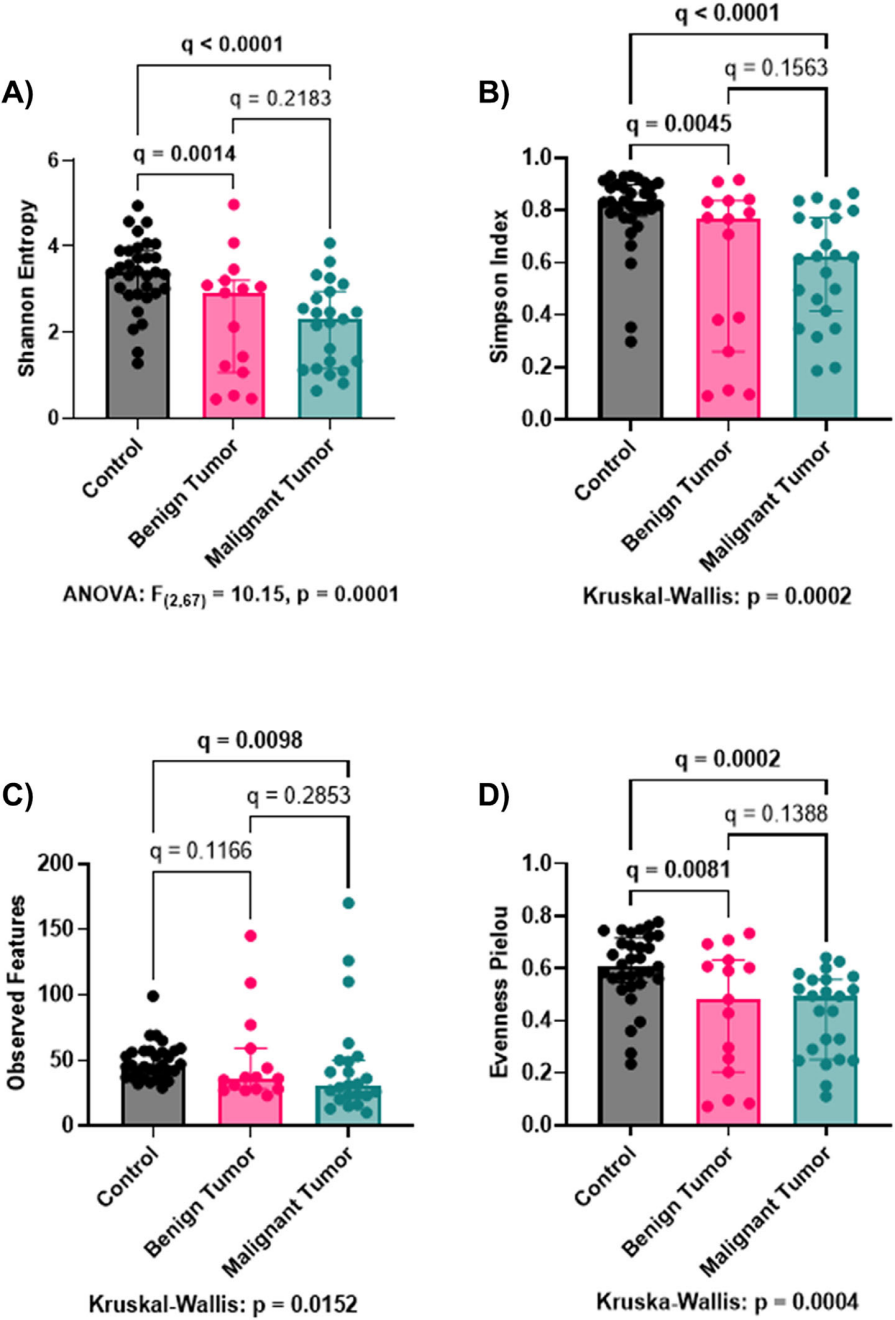
Tumor groups exhibit lower sinonasal microbial diversity across multiple indices. Alpha‐diversity indices are calculated for the sinonasal cavity within each participant, revealing significant differences between groups (ANOVA or Kruskal–Wallis test, *p* < 0.05) across all four metrics: (A) Shannon Entropy, (B) Simpson Index, (C) Observed Features, (D) Pielou's Evenness, and multiple group comparisons showed significantly lower Shannon Entropy, Simpson Index, and Pielou's Evenness in both benign tumor (BT) and malignant tumor (MT) participants compared to controls (*q* < 0.05), while Observed Features is significantly reduced only in MT participants (*q* = 0.0098). Median and interquartile ranges are shown. Black = control; pink = benign tumor; and green = malignant tumor.

Alpha‐diversity indices were calculated for the sinonasal cavity within each participant, revealing significant differences (ANOVA or Kruskal–Wallis, *p* < 0.05) between the groups across all four indices (Figure 1A–D). Notably, multiple group comparisons indicated that the BT and MT participants had significantly lower alpha‐diversity indices of Shannon Entropy, Simpson Index, and Pielou's Evenness compared to controls (*q* < 0.05), whereas Observed Features were significantly lower only in MT participants compared to controls (*q* = 0.0098). The Shannon Entropy was lowest in the MT group (median 2.31, interquartile range [IQR]: 1.16–2.95), followed by the BT group (median 2.92, IQR: 1.07–3.21), and highest in controls (median 3.39, IQR: 2.89–3.90). Similar outcomes were observed for the Simpson Index (controls: median 0.83, IQR: 0.78–0.90; BT: median 0.77, IQR: 0.26‐0.84; MT: median 0.62, IQR: 0.42–0.77), Observed Features (controls: median 45.50, IQR: 37–56.75; BT: median 36, IQR: 28–59; MT: median 31, IQR: 23–50), and Pielou's Evenness (controls: median 0.61, IQR: 0.54–0.72; BT: median 0.48, IQR: 0.20–0.63; MT: median 0.49, IQR: 0.25–0.55) (Figure 1B–D). The lower Shannon diversity index in the BT and MT groups indicates reduced species variation and evenness, meaning these participants’ sinonasal microbial communities are less diverse and more dominated by fewer bacterial taxa compared to controls.

**FIGURE 2:**
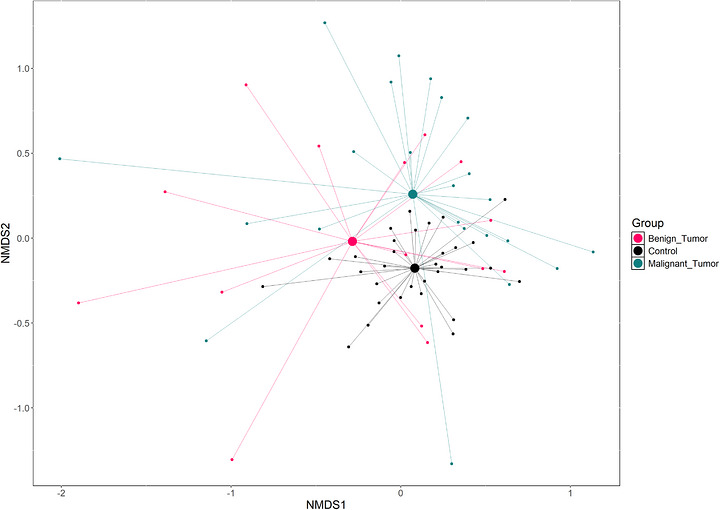
Distinct sinonasal microbial community structures in tumor groups compared to control patients. Beta‐diversity microbial community differences between groups are assessed with PERMANOVA on Aitchison distances, indicating significantly different compositions between groups. Centroid‐based nonmetric multi‐dimensional scaling (NMDS) plots depict microbial community differences. Control to benign tumor (PERMANOVA: *F* = 9.472, *q* = 0.0003), control to malignant tumor (PERMANOVA: *F* = 21.331, *q* = 0.0003), and benign tumor to malignant tumor (PERMANOVA: *F* = 1.418, *q* = 0.2475). Black = control; pink = benign tumor; and green = malignant tumor.

Beta‐diversity analyses revealed significant differences in the overall sinonasal microbial community compositions between controls compared to both BT and MT participants (PERMANOVA: *q* = 0.0003; Figure 2). Notably, the overall microbial compositions of the BT and MT groups did not differ significantly (PERMANOVA: *q* = 0.2475). Among the three groups, the MT sinonasal participants showed significantly greater variability within their group compared to controls (PERMDISP: *q* = 0.0399), while no significant within‐group dispersion differences were observed in other comparisons.

**FIGURE 3:**
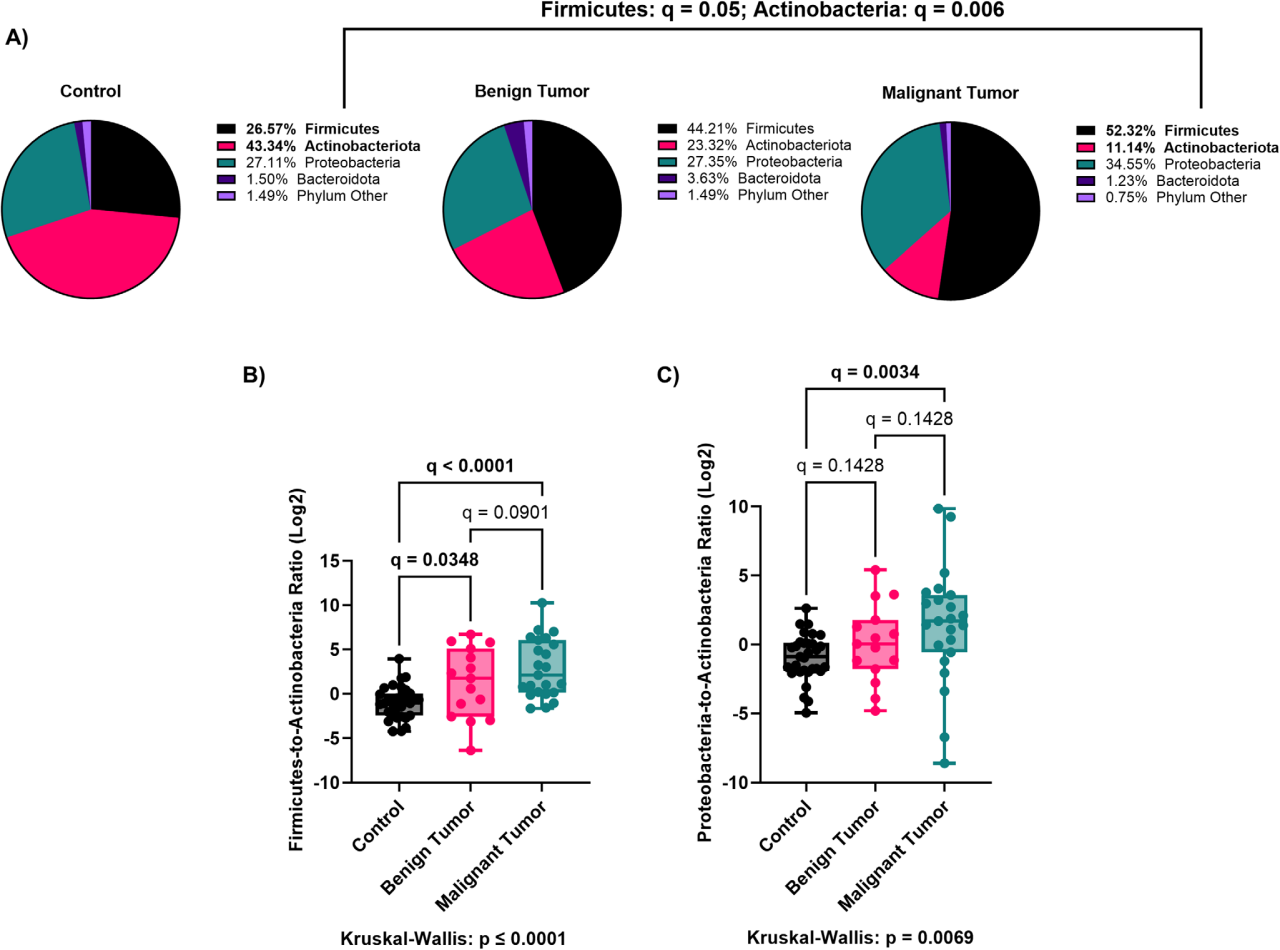
Phylum‐level alterations in sinonasal microbial compositions across groups. (A) Relative abundance profiling at the phylum level reveals distinct differences in sinonasal bacterial communities between groups. MT participants exhibit a significantly increased abundance of Firmicutes and decreased Actinobacteria compared to controls (CLR‐KW; *q* < 0.05 and *q* = 0.006, respectively). (B) The log2 ratio of Firmicutes‐to‐Actinobacteria is significantly elevated in both BT (*q* = 0.0348) and MT (*q* < 0.0001) participants compared to controls. (C) The putative proinflammatory Proteobacteria‐to‐Actinobacteria log2 ratio is significantly increased in the MT group relative to controls (*q* = 0.0034). BT, benign tumor; MT, malignant tumor.

Sinonasal cavity microbial community profiling revealed distinct differences in bacterial community compositions between groups at the taxonomic phylum level (Figure 3A). The sinonasal cavity of MT participants displayed increased relative abundance of Firmicutes (CLR‐KW: *q* < 0.05) and a decrease of Actinobacteria (CLR‐KW: *q* = 0.006) compared to controls. Further analysis revealed that both BT (*q* = 0.034) and MT (*q* < 0.0001) sinonasal cavities had a significantly higher Firmicutes‐to‐Actinobacteria ratio compared to controls (Figure 3B). Additionally, the ratio of the Proteobacteria‐to‐Actinobacteria (a proxy for proinflammatory and commensal taxa, respectively) significantly increased (*q* = 0.034) in the MT group relative to controls (Figure 3C).

**FIGURE 4:**
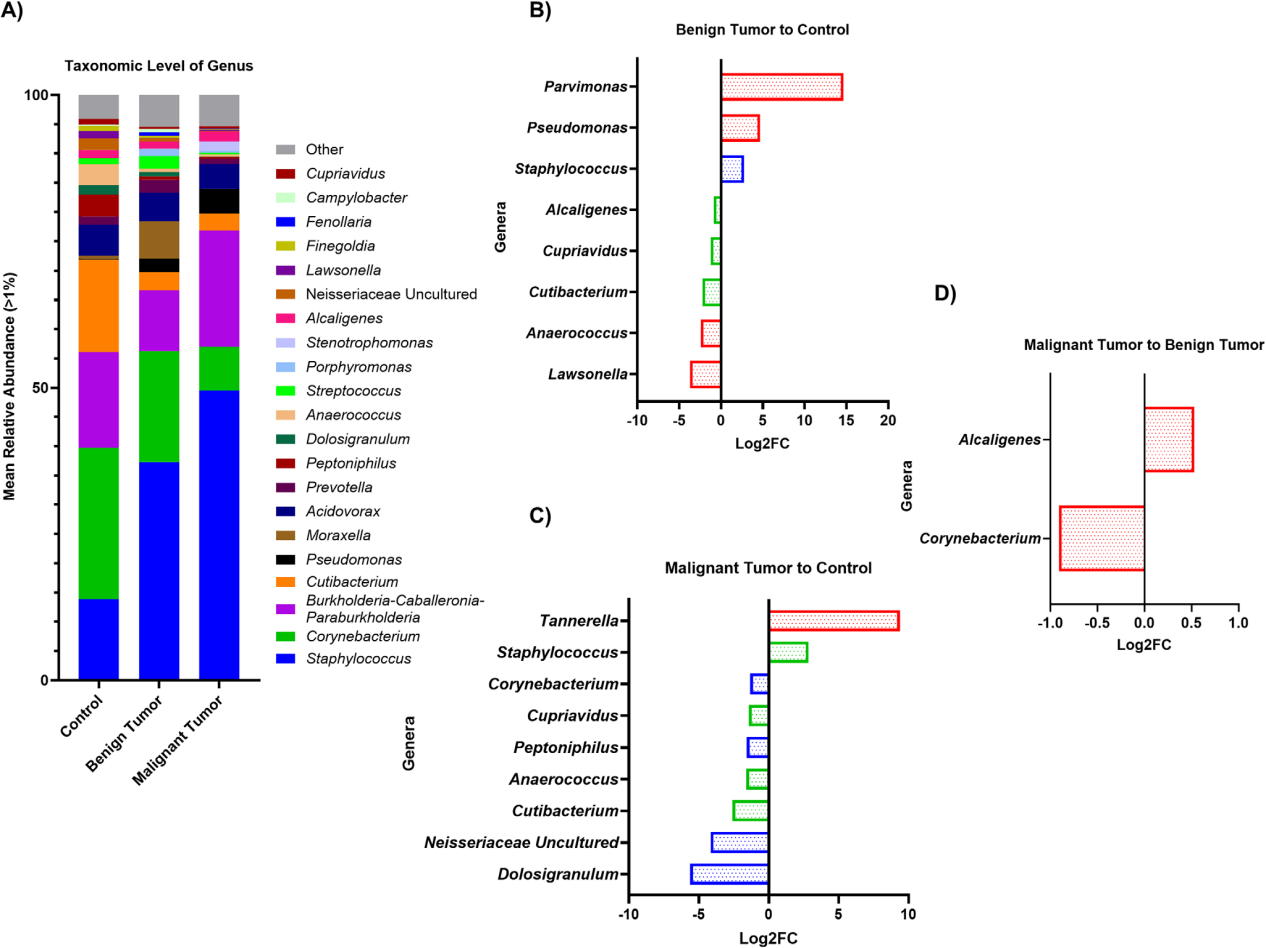
Genus‐level shifts in sinonasal microbial compositions across groups. (A) Stacked bar plot displaying the mean relative abundance of microbial genera (>1%) in sinonasal swabs between groups. Notable shifts in microbial composition are observed between groups. Genera contributing less than 1% of total abundance are grouped under “Other.” Genera identified as only significantly differentially abundant (red bar), significant plus machine‐learning feature of importance (green; Table 3), or only a feature of importance (nonsignificant) (blue; Table 3) are shown with their log2 fold‐change values in comparisons: (B) benign tumor to controls, (C) malignant tumor to controls, and (D) malignant tumor to benign tumor.

Sinonasal cavity microbial profiles at the genus level showed dysbiotic characteristics between controls and tumor groups. A machine‐learning‐based feature (Boruta) selection identified key genera of importance driving composition alterations (Table 2), accompanied by differential abundance analysis of individual taxa between groups (Figure 4A–D) and individuals (Figure S1). The BT participants showed a significant increase in relative abundance of bacteria from the putative Gram‐negative proinflammatory genera *Parvimona*s (CLR‐KW: *p* = 0.036) and *Pseudomonas* (CLR‐KW: *p* = 0.012), paired with significant losses of sinonasal anti‐inflammatory *Cutibacterium* (CLR‐KW: *p* = 0.011) and commensal genera *Anaerococcus* (CLR‐KW: *p* = 0.021), *Lawsonella* (CLR‐KW: *p* = 0.021)*, Alcaligenes* (CLR‐KW: *p* = 0.006) and *Cupriavidus* (CLR‐KW: *p* = 0.025) (Figure 4A,B) [25, 26, 27].

**TABLE 2:** Machine learning analysis identifies genera of importance distinguishing sinonasal swab samples from control and tumor groups.

Taxonomic level of genus	Number of important genera features identified
**Control vs. benign tumor**	
*Staphylococcus*	1
*Cutibacterium*	2
*Cupriavidus*	3
*Alcaligenes*	4
**Control vs. malignant tumor**	
*Staphylococcus*	1
*Cutibacterium*	2
*Cupriavidus*	3
*Corynebacterium*	4
*Anaerococcus*	5
*Dolosigranulum*	6
*Peptoniphilus*	7
*Neisseriaceae* uncultured	8
**Benign tumor vs. malignant tumor**	
No features of importance	−

*Note*: Distinguished signatures of featured genera of importance between control and sinonasal tumor samples were identified using the machine learning algorithm *Boruta*. Genera that encompassed 90% of the overall microbial composition were included in the analysis. Control (*n* = 32), benign tumors (*n* = 15), and malignant tumors (*n* = 23).

Compared to controls, MT participants displayed increased relative abundance of putative proinflammatory genera *Staphylococcus* (CLR‐KW: *q* = 0.012) and *Tannerella* (CLR‐KW: *p* = 0.037). Like BT participants, the MT participants also exhibited significant decreased relative abundances of beneficial sinonasal anti‐inflammatory *Cutibacterium* (CLR‐KW: *q* = 0.002) and commensal bacterial genera *Anaerococcus* (CLR‐KW: *q* = 0.016) and *Cupriavidus* (CLR‐KW: *q* = 0.021) (Figure 4A,C).

Finally, the comparison between BT and MT participants revealed few differentially abundant taxa (Figure 4A,D), with MT sinonasal cavity swab showing significantly increased relative abundance of Gram‐negative opportunistic inflammatory *Alcaligenes* (CLR‐KW: *p* = 0.049) and lower relative abundances of beneficial sinonasal bacteria from the genus *Corynebacterium* (CLR‐KW: *p* = 0.049).

## 4 Discussion

In this prospective, cross‐sectional, observational study of the sinonasal microbiota, we demonstrate that both BT and MT sinonasal tumor samples are associated with significant microbial dysbiosis, characterized by significantly lower microbial diversity, distinct alterations in microbial community structure, decreased abundance of beneficial microbes, and increased abundance of potentially pathogenic bacteria. These findings provide the first high‐resolution sequencing‐based evidence of tumor‐associated nasal microbiota changes and suggest a potential role for microbial communities in sinonasal tumor biology.

Our study showed that MT samples had reduced microbial alpha‐diversity, which aligns with patterns previously observed in other mucosal cancers, including colorectal and oropharyngeal malignancies, where loss of diversity often precedes or parallels neoplastic transformation [28, 29]. The intermediate loss in microbial diversity within BT samples may reflect localized tissue remodeling or immune perturbations that are less severe than those induced by malignancy. Greater within‐group dispersion in the MT samples further supports the concept of microbial community instability as a feature of the malignant tumor microenvironment [30].

The significantly elevated Firmicutes‐to‐Actinobacteria ratio observed in both BT and MT groups underscores a major shift in sinonasal microbial community structure associated with tumor presence. This significant imbalance, especially in MT samples, suggests a disruption of the healthy sinonasal microbial ecosystem, as the relative abundance of Actinobacteria such as *Corynebacterium* and *Cutibacterium*, which can serve beneficial, anti‐inflammatory roles, was significantly decreased [31]. Conversely, the relative abundances of putative proinflammatory/opportunistic pathogens from the phylum Firmicutes, such as *Parvimonas* and *Staphylococcus*, were increased. Furthermore, the significant increase in the Proteobacteria‐to‐Actinobacteria ratio in MT samples highlights a potential proinflammatory microbial signature, which may contribute to tumor development or represent a response to tumor development. Together, these phylum‐level altered ratios provide evidence that sinonasal microbiota dysbiosis may play a role in tumor pathogenesis.

Tumor‐related changes in local tissue architecture, vascularization, immune surveillance, and oxygen tension may all contribute to sinonasal microbial dysbiosis [3]. Compared to controls, putative proinflammatory genera *Parvimonas*, *Pseudomonas*, and *Staphylococcus* were higher in BT samples, wherein *Staphylococcus* was then further increased along with *Tannerella* in MT samples. The increased relative abundance of *Staphylococcus*, which may be attributed to the opportunistic pathogen *S. aureus*, in both BT and MT samples, may indicate an early‐stage to late‐stage microbial dysbiotic imbalance, wherein commensal suppression progresses, where pathogen dominance is more extreme in malignant tumors. This progressive dysbiotic microbial shift could explain why few significant differences were observed between BT and MT microbiota, despite both diverging markedly from controls.

Furthermore, hypoxic niches within tumors may favor the growth of obligate anaerobes such as *Parvimonas*, while facultatively anaerobic organisms like *Pseudomonas* may adapt to low‐oxygen conditions through metabolic pathways. Bacteria from the genus *Parvimonas* have been theorized to contribute to tumorigenesis in colorectal carcinoma as well as nasopharyngeal carcinoma [32, 33, 34].

Concurrently, chronic inflammation and mucosal barrier breakdown may promote overgrowth or increased activity of commensal organisms like *Staphylococcus*, as well as opportunistic taxa including *Tannerella*. While *Staphylococcus* is a common organism of healthy sinonasal microbiota, its relative abundance and pathogenic potential may increase in dysbiotic or inflamed mucosal environments [35]. From a tissue pathology perspective, sinonasal MTs are distinct from BTs, with more severe local destruction at a cellular level [36, 37]. Consistent with this alteration of host physiology, we observed a stepwise proinflammatory microbiota shift between BT and MT samples. Whether these microbial shifts are causative or consequential of tumor development remains unclear, but the observed patterns support the plausibility of bidirectional interactions between tumor and microbiome.

Within this broader dysbiotic trajectory, enrichment of *Pseudomonas* remains notable, as this pathogen is well known for its virulence, biofilm formation, and resistance to host immunity and antibiotics [38]. In pulmonary and cutaneous systems, *Pseudomonas* has been shown to produce toxins and inflammatory mediators that modulate host immunity and may impair epithelial repair [38]. In the context of sinonasal tumors, its presence may reflect an opportunistic colonization of immunocompromised tissue or, more provocatively, may suggest a role in tumor promotion through mechanisms such as chronic inflammation, genotoxicity, or immune modulation. Prior studies have linked *Pseudomonas* and *Staphylococcus* to worse outcomes in CRS and postoperative infections, but their potential relationship to sinonasal malignancy has not been described [39, 40]. Similarly, bacteria from the genus *Tannerella* have been linked to periodontitis and associated odontogenic rhinosinusitis [41]. These associations support the hypothesis that these genera may serve as markers or potential therapeutic targets in the context of microbial dybiosis relating to pathologic disease states.

Our findings suggest that these increases in the relative abundances of *Parvimonas*, *Staphylococcus*, *Tannerella*, and *Pseudomonas* may contribute to, or act as markers of, intensified inflammatory changes observed in MT, warranting further mechanistic investigation.

The marked depletion of bacteria from the genera *Cutibacterium* and *Corynebacterium* in both BT and MT samples is noteworthy. These taxa are considered keystone, anti‐inflammatory bacteria of a healthy nasal microbiome, and can play a protective role by occupying ecological niches and inhibiting overgrowth of pathobionts such as *Staphylococcus, Tannerella, Parvimonas*, and *Pseudomonas* [42]. In vitro studies have shown that *Corynebacterium* produces free fatty acids that inhibit pathogen growth, while *Cutibacterium* may modulate local immune responses [43, 44]. Their lower relative abundance in tumor‐bearing patients may reflect competitive exclusion by pathogenic organisms or local environmental changes (e.g., pH, oxygenation) that suppress commensal viability.

These results highlight the potential for microbiome profiling as a diagnostic or prognostic adjunct in sinonasal tumors. Further studies differentiating between microbial signatures are necessary as they may aid in distinguishing benign from malignant lesions. They may also help differentiate between the different types of benign, as well as malignant tumors, and may have a role to play, particularly in ambiguous or difficult‐to‐biopsy cases. Future studies with large sample sizes and inclusion of diverse tumor subtypes are needed to validate these findings and fully explore the diagnostic potential of microbiome profiling in sinonasal neoplasms.

However, important questions remain. First, the cross‐sectional nature of our study precludes causal inference. Future studies that investigate the functional roles and pathogenic mechanisms of these bacteria, along with their links to tumorigenesis, are essential as a next step. If causative relationships are established similar to gastrointestinal tumors, microbiota‐modulating interventions such as probiotics, antibiotics, or topical therapies could be developed for reducing risk, treating disease, or preventing recurrence. Longitudinal sampling from premalignant, benign, and malignant states would help clarify whether microbial changes precede or follow tumor development. Second, while short‐read 16S rRNA gene amplicon sequencing provides broad taxonomic coverage, it lacks complete species‐level resolution and metagenomic functional gene/pathway inference. Future studies employing full‐length 16S rRNA gene amplicon sequencing, whole‐metagenome sequencing, or metabolomics could elucidate microbial activity and host–microbe interactions in greater detail.

This study has several limitations. While the sample size was adequate to detect group‐level differences, larger cohorts would improve statistical power for subgroup analyses (e.g., tumor subtype, location, treatment history). Although we controlled recent antibiotic exposure, other unmeasured variables, such as diet, comorbidities, and environmental exposures such as tobacco use and prior radiation exposure, may have influenced microbial composition. The relatively small cohort size also limited power for subgroup analyses. Viral factors such as HPV and EBV were not evaluated, which limits the ability to account for potential viral–microbial interactions that may contribute to tumor biology. Despite these limitations, this study provides important foundational data on tumor‐associated dysbiosis in the sinonasal tract. To our knowledge, this is the first study to identify specific bacterial taxa associated with sinonasal tumors, including enrichment of several proinflammatory ratios and taxa and the depletion of protective, gatekeeping commensal bacteria.

## 5 Conclusion

This study highlights significant dysbiosis in the sinonasal microbiota of MT samples, characterized by a pronounced loss of commensal taxa, with similar but fewer alterations observed in BT samples. These findings underscore the need for future research to determine whether these microbial alterations play a causal role in tumor development or are secondary to tumor presence. Elucidating the functional significance of these microbial changes may reveal novel diagnostic biomarkers or therapeutic targets in sinonasal oncology.

## Author Contributions

*Conceptualization*: M.M., P.S.B. *Investigation*: E.P., P.A.E., G.D.S., S.K., D.M., S.J.G, P.F., P.P., B.A.T, M.M., P.S.B. *Data curation and formal analysis*: P.A.E., S.K., D.M., E.P., L.K., S.J.G., A.N. *Writing – original draft preparation*: E.P., P.A.E. *Writing – review and editing*: P.A.E., G.D.S., S.K., D.M., E.P., L.K., S.J.G., A.N., P.F., P.P., B.A.T., M.M., P.S.B. All authors have read and agreed to the published version of the manuscript.

## Funding

These microbiome analyses were funded through the Miller Family Foundation philanthropic support to the Sinus, Allergy, and Asthma Center.

## Conflicts of Interest

The authors declare no conflicts of interest.

## Supporting information




**Supporting Table S1**: alr70123‐sup‐0001‐TableS1.xlsx.

**Figure S1**: Genus‐level shifts in sinonasal microbial compositions across individual participants. (A) Stacked bar plot displaying the mean relative abundance of microbial genera (>1%) in sinonasal swabs among individuals and across the overall group assignments. Notable shifts in microbial composition are observed between individuals. Genera contributing less than 1% of total abundance are grouped under “Other.”

## Data Availability

The data presented in this article are openly available in the National Center for Biotechnology Information (NCBI) BioProject database under accession number PRJNA1308578. The NCBI Biosample and Sequence Read Archive (SRA) database table is shown (Table S1).
