# Supplementary figures and images for: Altered Nasal Microbiota in Sinonasal Tumors: A Comparative Analysis of Malignant and Benign Sinonasal Tumors
Source: Int Forum Allergy Rhinol. 2026 Feb 20;16(8):778–87. doi: 10.1002/alr.70123 (PMC13432638; doi:10.1002/alr.70123)

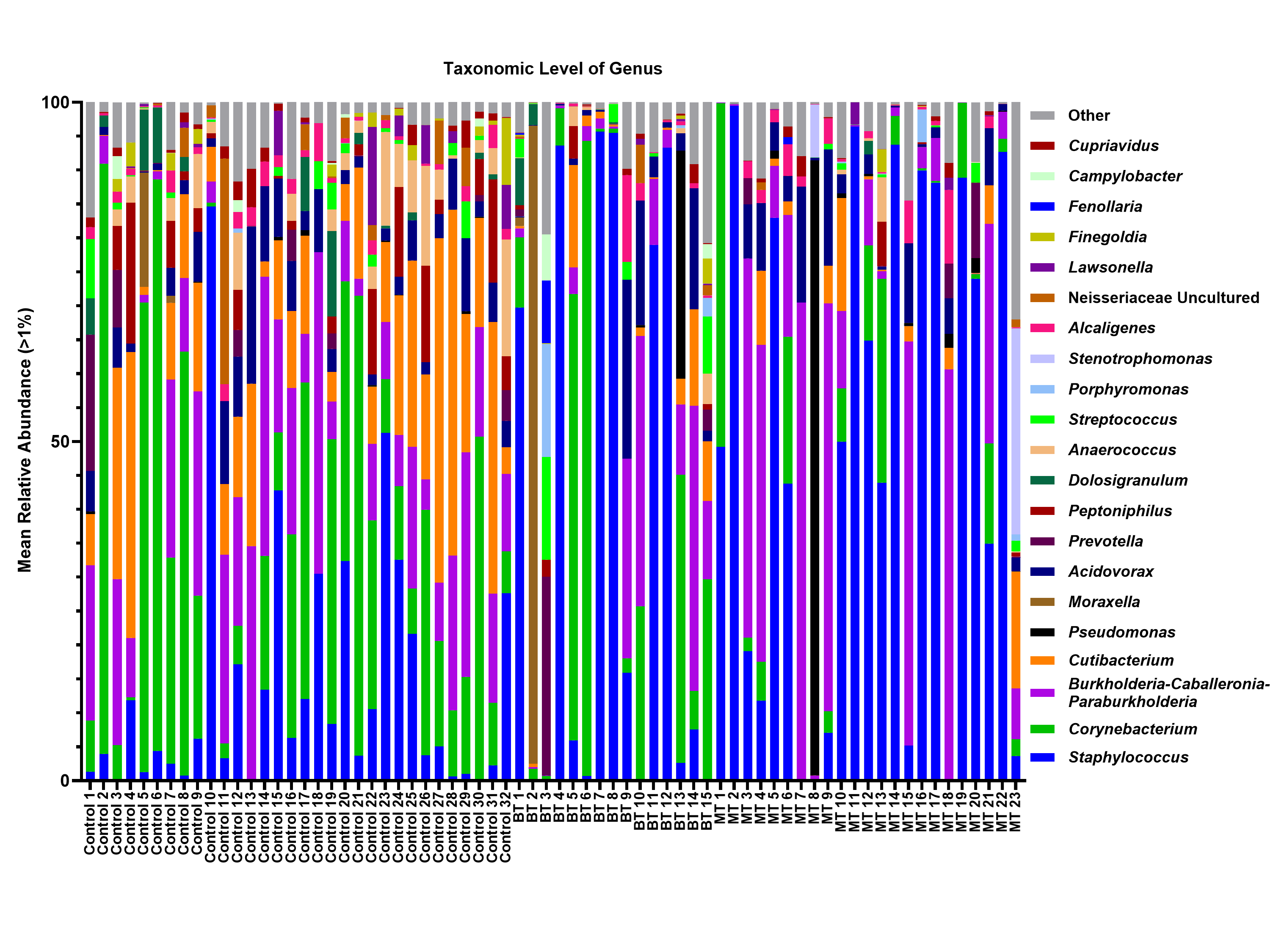

Supplement: Supplementary file 2 — Figure S1: Genus‐level shifts in sinonasal microbial compositions across individual participants. (A) Stacked bar plot displaying the mean relative abundance of microbial genera (>1%) in sinonasal swabs among individuals and across the overall group assignments. Notable shifts in microbial composition are observed between individuals. Genera contributing less than 1% of total abundance are grouped under “Other.” [file ALR-16-778-s001.TIF]
